# Vitamin D and family history of hypertension in relation to hypertension status among college students

**DOI:** 10.1038/s41371-021-00577-6

**Published:** 2021-07-20

**Authors:** Yendelela L. Cuffee, Ming Wang, Nathaniel R. Geyer, Sangeeta Saxena, Suzanne Akuley, Lenette Jones, Robin Taylor Wilson

**Affiliations:** 1 Epidemiology Program, College of Health Sciences, University of Delaware, Newark, DE USA; 2 Department of Public Health Sciences, College of Medicine, Pennsylvania State University, Hershey, PA USA; 3 University of Michigan, School of Nursing, Ann Arbor, MI USA; 4 Department of Epidemiology and Biostatistics, College of Public Health, Temple University, Philadelphia, PA USA

**Keywords:** Hypertension, Risk factors

## Abstract

Hypertension and vitamin D concentrations have heritable components, although these factors remain uninvestigated in young adults. The objective of this study was to investigate hypertension risk among young adults with respect to family history of hypertension, adjusting for vitamin D status. Resting blood pressure (BP) was measured in 398 individuals aged 18–35 and classified according to the 2017 American Heart Association criteria. Plasma vitamin D metabolite (25(OH)D_3_; 24,25(OH)_2_D_3_; 1,25(OH)_2_D_3_) concentrations were determined using liquid chromatography tandem mass spectrometry (LC-MS/MS). Stepwise logistic regression was used to select covariates. Participants' mean age was 21, 30.3% had hypertension, and nearly all unaware of their hypertensive status (90.7%). Compared with no parental history, the adjusted odds ratio (AOR) for hypertension was elevated among participants with two parents having hypertension (AOR = 4.5, 95% CI: 1.70–11.76), adjusting for sex, body mass index, physical activity, and plasma 25(OH)D_3_. Results for systolic hypertension (SH) were similar but more extreme (two parents AOR = 7.1, 95% CI: 2.82, 17.66), although dihydroxy metabolites (1,25(OH)_2_D_3_ and 24,25(OH)_2_D_3_) were significant. There was a strong, independent association with dual parental history and hypertension status, regardless of vitamin D status. Hypertension was prevalent in nearly one-third of the sample and underscores the need for targeted prevention for young adults.

## Introduction

The 2017 Guidelines for the Prevention, Detection, Evaluation and Management of High Blood Pressure from the American College of Cardiology (ACC) and American Heart Association (AHA) reported 103.3 million adults have hypertension [1]. In spite of the increasing prevalence of hypertension in the United States among young adults, a majority of programs and initiatives to reduce high blood pressure have been targeted at middle aged and older adults. The growing prevalence of hypertension among young adults is typically attributed to lifestyle factors such as diet and exercise [1]. Despite the risk of developing hypertension, young adults are often unaware of their hypertension status and less likely to have controlled hypertension. Findings from the 2019 Heart Disease and Stroke Statistics indicated that among 20–39-year olds approximately 42% were aware of their diagnosis, 21% were being treated, and 10% had controlled hypertension. The findings indicated the rates of awareness of hypertension status were lowest among young adults (20–39) compared to other age groups [1, 2]. To appropriately diagnose, treat, and reduce the rates of incident hypertension among young people, it is essential to have a comprehensive understanding of the risk factors for elevated and high blood pressure within this age group.

Family history of hypertension is a risk factor for hypertension among children and young adults [3, 4]. A family history of high blood pressure is associated with an increased risk of developing hypertension, earlier onset hypertension, and adverse outcomes from hypertension [4]. Findings from the Framingham Heart Study (FHS) highlighted the importance of the familial history of high blood pressure. The results from the FHS indicated the odds of having hypertension were three times greater for offspring that had two parents with early onset of hypertension [4].

There is a growing interest in examining vitamin D biomarkers and the risk for cardiovascular disease [5, 6]. Low plasma vitamin D levels (i.e., <60 nmol/L) are a risk factor for developing cardiovascular diseases such as stroke and myocardial infarction [7]. Vitamin D depletion activates the renin-angiotensin-aldosterone system, and the activation of this system increases the risk of developing arterial hypertension [8, 9]. There are over 50 known biomarkers of vitamin D, but 25(OH)D_3_ [10, 11], is most commonly used to assess vitamin D; and has been used for multiple cross-sectional and retrospective case-control studies [7]. Few studies have examined the relationship between vitamin D and family history of cardiovascular disease. Khalili et al conducted a study of 139 adults after acute myocardial infarction, to examine vitamin D concentration and cardiovascular disease, their findings revealed low 25(OH)D levels were associated with a positive family history of CVD [12]. Circulating blood concentrations of vitamin D may also have a direct role in metabolic syndrome, hypertension, and insulin resistance [13, 14], but the association among young adults remains unclear.

To date, few studies have explored potential risk factors for developing hypertension such as plasma vitamin D levels and family history among young adults. Given the low rates of hypertension awareness, treatment, and control among young adults, identifying risk factors for the elevated and high blood pressure may be helpful for raising awareness about hypertension and hypertension management. Early identification of risk factors can provide access to primary prevention efforts, such as lifestyle and behavioral change, and provide opportunities to tailor hypertension interventions for young adults, which may help to reduce the risk of cardiovascular disease later in life [15]. The objective of this study was to examine the prevalence of high and elevated blood pressure among young adults and explore family history and plasma vitamin D as potential risk factors for hypertension.

## Subjects and methods

### Study population

The data for this study were obtained during multiple data collection periods of the Sunlight Nutrition, Skin, and Human Ancestry related to vitamin D Exposure and Synthesis (SuNSHADES) Study, as previously described [16]. SuNSHADES was conducted during non-summer months in three waves as follows: Wave 1—February 2006–February 2007; Wave 2—February–May 2009, and Wave 3—November 2013–April 2016. Study participants were recruited from Penn State University using institutional list serves, newspapers, and personal interactions. All study participants consented to participate in the study. Participants with hypotension, diabetes, currently taking antihypertensives, steroids, race/ethnicity, or with missing information on vitamin D biomarker concentrations were excluded from the current study, in total 65 participants were excluded from the study. During a single clinic visit to the Penn State Clinical Research Center, participants completed a questionnaire which included gender, age, race/ethnicity, and family history of high blood pressure, BMI, and latitude at the city of birth. Height and weight were measured using the National Health and Nutrition Examination Survey (NHANES) protocol. Standing height was measured in meters using a stadiometer with a fixed vertical backboard and an adjustable head piece. Participants were correctly positioned and weighed in kilograms using a digital weight scale. Individuals were encouraged to bring in all medications for recording purposes. The dose for oral contraceptives (ethinyl estradiol ug/day), hypertensive medication use and antidepressant use was classified using the Prescriber’s Digital Reference (www.pdr.net). At the time of the visit, all individuals were made verbally aware of their blood pressure status by the attending study nurse and provided hardcopy materials on high blood pressure risks from the Centers for Disease Control and Prevention. All waves of the study were approved by the Pennsylvania State College of Medicine IRB Protocol Number 22524 (Waves 1 and 2) and 43010 (Wave 3).

### Outcome measurements

Blood pressure was measured following the NHANES protocol by clinical research nurses trained in the protocol and were consistent across each time period data were collected [17]. In brief, two consecutive blood pressure measurements were taken after the participant was seated for 5 min. Using the arithmetic mean of systolic and diastolic, four categories of blood pressure were determined: normal (systolic < 120 mmHg and diastolic <80 mmHg), elevated (systolic 120–129 mmHg and diastolic <80 mmHg), hypertension stage 1 (systolic 130–139 mmHg or diastolic 80–89 mmHg) and hypertension stage 2 (systolic ≥ 140 mmHg or diastolic ≥90 mmHg) in accordance with the 2017 ACC/AHA/AAPA/ABC/ACPM/AGS/APhA/ASH/ASPC/NMA/PCNA Guideline for the Prevention, Detection, Evaluation, and Management of High Blood Pressure in Adults [18–20].

### Vitamin D biomarkers

Four plasma vitamin D biomarkers 25(OH)D_3_, 25(OH)D_2_, 24,25(OH)_2_D_3_, and 1,25(OH)_2_D_3_ were measured using liquid chromatography with tandem mass spectrometry (LC/MS-MS) as described previously [16]. The lower limit of quantitation (LLOQ) for 25(OH)D_3_, 25(OH)D_2_, 24,25(OH)_2_D_3_ metabolites was 10 pg/mL, with a linear range of 1–100 ng/mL for 25(OH)D_2_ and 25(OH)D_3_ and 0.1–10 ng/mL for 24,25(OH)_2_D_3_ [16]. For 1,25(OH)_2_D_3_, the lower limit of quantitation (LLOQ) is 5 pg/mL with a linear range of 5 to 1000 pg/mL (Dai, 2015). The percent coefficient of variation, assayed in 10 repeated samples of pooled plasma from healthy individuals on separate days, was 2.7, 12.2, and 6.3 for 25(OH)D_3_, 25(OH)D_2_, 24,25(OH)_2_D_3_, respectively. Because 25(OH)D_2_ is predominantly associated with environmental (dietary and supplement intake) and not genetic factors; therefore, we did not include 25(OH)D_2_ in the analysis.

### Genotyping and genetic ancestry

DNA was extracted from whole blood in tubes with ethylenediaminetetraacetic acid using aQIAamp DNAMini Kit (Qiagen Sciences, Germantown, Maryland) and stored at −80 °C. A panel of 112 ancestry-informative markers was used to estimate the proportion of West-African ancestry and proportion of European ancestry, using the STRUCTURE algorithm and frequencies inHapMapYRI (WestAfrican) and CEU(European) trios [21, 22]. This panel exhibits high agreement with other ancestry panels (concordance correlation coefficient = 0.97). Genotypes were determined using the Illumina BeadXpress assay (Illumina Inc., San Diego, California). Genotyping cluster algorithm outputs were visually inspected, and genotyping calls were adjudicated by laboratory personnel blinded to the vitamin D status and study characteristics of participants. Samples and assays with >10% unreadable genotyping calls were excluded from the analysis.

### Statistical methods

BMI was calculated as weight in kilograms over height in meters squared (kg/m^2^) and classified according to the World Health Organization (2000) BMI Classification [16]. Quartiles of vitamin D biomarkers were determined and classified according to quartile cut-off values among normotensive participants. Participants with missing BMI, physical activity, and/or smoking status were excluded from the analysis. Two-tailed student’s t and chi-square tests were used to test for (unadjusted) bivariate differences in continuous and categorical variables, respectively. Bivariate tests for trend in categorical variables with respect to normotensive, elevated blood pressure and hypertensive status were calculated using the Mantel–Haenszel chi-square. Bivariate tests for trend among quantitative variables with respect to normotensive, elevated blood pressure, and hypertensive status were calculated using linear regression (SAS Proc REG).

### Multiple regression model

Potential confounding variables were assessed using stepwise selection in logistic regression for (1) the odds of hypertension (versus normotension), i.e., excluding those with elevated blood pressure; (2) the odds high systolic blood pressure; and (3) the odds of high diastolic blood pressure. Those variables with a bivariate *p-*value of 0.150 or less were placed into a stepwise selection model (*p* = 0.150 to enter, *p* = 0.100 to stay in the model). For purposes of comparison, the same adjustment variables found in association with hypertension were also used in the multiple regression model of the odds of elevated blood pressure (versus normotension). In order to assess possibility of collinearity of the adjustment variables, Spearman Correlation coefficients were calculated for all variables included in the final models. All *p-*values were two-sided, *p* = ≤ 0.05 was considered statistically significant, and all analyses were performed using SAS 9.4.

## Results

### Bivariate associations

After applying exclusion criteria, the analysis included 396 young adults from all three waves of the study (Supplemental Fig. 1). In bivariate analyses, elevated blood pressure status and hypertension status were significantly associated with sex, parental history of hypertension (both parents), self-reported history or hypertension, parental history of diabetes (neither parent), and body mass index (all *p* = < 0.05, Table 1). Vitamin D biomarkers (1,25(OH)_2_D_3_ and 24,25(OH)_2_D_3_) were negatively associated both with elevated blood pressure and hypertension status (all *p* = <0.010, Table 2). Both 1,25(OH)_2_D_3_ and 24,25(OH)_2_D_3_, monotonically decreased from normotensive, elevated blood pressure, and hypertensive group status, although this was not the case for 25(OH)D_3_ which had similar (and lower) values for elevated and hypertensive groups (59.4 nmol/L and 60.2 nmol/L, respectively) when compared with normotensive individuals. We did not detect a statistically significant association between West African genetic ancestry proportion and elevated blood pressure/hypertension status (p*-*trend = 0.337). Individuals classified with hypertension had a mean systolic blood pressure of 123.8 (±10.3) mmHg and a mean diastolic blood pressure of 83.6 (±5.4) mmHg (Table 2). Neither paternal nor maternal history of kidney stones, obesity, or a thyroid disorder were associated with the likelihood of hypertension in bivariate tests of association (data not shown).

**Table 1:** Characteristics of study participants by blood pressure status, adults ages 18–35, Centre County Pennsylvania, 2006–2016.

	Normotensive *N* (%)	Elevated *N* (%)	Hypertensive Stage I and II N (%)	Total *N* (%)	*p*-trend
Total	238 (100.0)	38 (100.0)	120 (100.0)	396 (100.0)	
Female	168 (70.6)	15 (39.5)	54 (45.0)	237 (59.9)	**<0.001**
Race/ethnicity					
White, non-hispanic	113 (47.5)	11 (29.0)	52 (43.3)	176 (44.4)	0.339
Black, non-hispanic	109 (45.8)	26 (68.4)	64 (53.3)	199 (50.3)	0.112
Hispanic/Latinx	16 (6.7)	1 (2.6)	4 (3.3)	21 (5.3)	0.154
Parental history of hypertension					
Neither parent	128 (53.8)	15 (39.5)	53 (44.2)	196 (49.5)	0.065
Both Parents	13 (5.5)	9 (23.7)	15 (12.5)	37 (9.3)	**0.013**
Mother Only	38 (16.0)	7 (18.4)	21 (16.7)	66 (16.4)	0.837
Father Only	54 (22.7)	6 (15.8)	29 (24.2)	90 (22.5)	0.840
Parent status unknown/missing	5 (2.1)	1 (2.6)	3 (2.5)	9 (2.3)	0.799
Self-reported hypertension					
Yes	7 (2.9)	0 (0.0)	12 (10.0)	19 (4.8)	**0.006**
No	227 (95.4)	38 (100.0)	107 (89.2)	372 (93.9)	**0.033**
Unsure/missing	4 (1.7)	0 (0.0)	1 (0.8)	5 (1.3)	0.452
Parental history of diabetes					
Neither parent	198 (83.2)	31 (81.6)	88 (73.3)	317 (80.1)	**0.031**
Both parents	1 (0.4)	0 (0.0)	2 (1.7)	3 (0.8)	–
Mother only	12 (5.0)	4 (10.5)	8 (6.7)	24 (6.1)	0.463
Father only	22 (9.2)	3 (7.9)	19 (15.8)	44 (11.1)	0.074
Parent status unknown/missing	5 (2.1)	0 (0.0)	3 (2.5)	8 (2.0)	–
Current smoking status					
Never	212 (89.1)	36 (94.7)	98 (81.7)	346 (87.4)	0.068
Former	18 (7.6)	2 (5.3)	13 (10.8)	33 (8.3)	0.331
Current	8 (3.4)	0 (0.0)	9 (7.4)	17 (4.3)	0.096
Physical activity frequency					
None	41 (17.6)	8 (21.1)	27 (22.5)	76 (19.2)	0.222
A few times per month	46 (19.3)	16 (42.1)	32 (26.7)	94 (23.7)	0.067
1–3 days per week	49 (20.6)	1 (2.6)	27 (22.5)	77 (19.4)	0.895
4–6 days per week	55 (23.1)	8 (21.1)	18 (15.0)	81 (20.5)	0.076
Every Day	47 (19.8)	5 (13.2)	16 (13.2)	68 (17.2)	0.114
Body mass index (BMI) class					
Underweight	7 (2.9)	0 (0.0)	2 (1.7)	9 (2.3)	–
Normal	170 (71.4)	21 (55.3)	49 (40.8)	240 (60.6)	**<0.001**
Overweight	47 (19.8)	8 (21.1)	38 (31.7)	93 (23.5)	**0.014**
Obese	14 (5.9)	9 (23.7)	31 (25.8)	54 (13.6)	**<0.001**
Alcohol use (>1 drink per week)	50 (21.0)	8 (21.1)	24 (19.8)	82 (20.6)	0.830
Antidepressant use	17 (7.1)	4 (10.5)	14 (11.7)	35 (8.8)	0.146
Coffee intake (>1 cup per week)	85 (35.7)	9 (23.7)	33 (27.5)	127 (32.1)	0.092
Oral contraceptive use (women)	56 (33.3)	5 (33.3)	19 (35.2)	80 (33.8)	0.809

^*^One individual with unknown smoking status reclassified as ‘never smoker’-cell sizes too small for *P*-value trend calculation.

**Table 2:** Mean West African genetic ancestry proportion, vitamin D biomarkers, days after summer solstice at blood draw, body mass index and blood pressure among young adults (ages 18–35), by hypertension stage, Centre County Pennsylvania, 2006–2016.

	Normotensive mean (SD)	Elevated blood pressure mean (SD)	Hypertensive (Stage I and II) mean (SD)	*p*-trend	Spearman’s *R*^2^
Age	21.3 (3.0)	21.5 (2.8)	21.5 (3.0)	0.575	<0.001
West African Genetic Ancestry Proportion	39.6 (34.8)	51.1 (32.5)	42.6 (35.6)	0.337	0.002
25(OH)D_3_ (nmol/L)	76.8 (54.7)	59.4 (35.2)	60.2 (32.0)	**<0.001**	0.027
24,25(OH)_2_D_3_ (nmol/L)	10.1 (10.0)	7.0 (5.9)	6.5 (5.6)	**<0.001**	0.039
1,25(OH)_2_D_3_ (pmol/L)	101.9 (38.4)	95.0 (44.5)	90.2 (29.1)	0.004	0.021
Days after Summer Solstice at blood draw	105.3 (53.0)	113.0 (67.9)	97.4 (41.4)	0.217	0.004
Body Mass Index	23.9 (3.9)	28.1 (6.9)	28.1 (7.0)	**<0.001**	0.119
Systolic blood pressure (mmHg)	108.5 (6.2)	122.7 (2.8)	123.8 (10.3)	**<0.001**	0.460
Diastolic blood pressure (mmHg)	70.9 (4.9)	72.1 (6.2)	83.6 (5.4)	**<0.001**	0.515
Ethinyl Estradiol ug/day (OC users only)	25.7 (8.6)	15.0 (5.0)	25.0 (6.7)	**0.486**	0.007

*IU* International Units, *SD* standard deviation, *kg* kilograms, *m*^*2*^ meters squared, *nmol* nanomoles, *L* liters, *mg* milligrams, *dL* deciliter, *BMI* body mass index (BMI), West African Ancestry, and Vitamin D Biomarkers *P*-values were log transformed for this analysis.

### Variable selection

The following variables with a bivariate *p-*value of 0.150 or less were entered into the stepwise selection models: coffee intake indicator variable for >1 cup per week), gender (female v. male), body mass index (as WHO BMI Class), physical activity (none/unknown, a few times per month, 1–3 days per week, 4–6 days per week, daily), smoking (never, former, current smoking), current antidepressant use (yes/no), current oral contraceptive (OC) use (yes/no), daily OC dose of ethinyl estradiol (ug/day, continuous), number of days after summer solstice at the time of blood draw (quartile in the entire study population), and parental history of diabetes (for both parents, mom only, dad only, and unknown). In the model of hypertension (versus normotension), stepwise selection retained female sex, body mass index (normal weight and obese indicators), physical activity (none), anti-depressant use (yes), and one vitamin D biomarker (25(OH)D_3_) as potential adjustment variables (all *p* = <0.100, hypertensive vs. normotensive). After inclusion of parental history of hypertension in the model, the variables for sex, body mass index, physical activity, and 25(OH)D_3_ remained statistically significant and were therefore retained in the final model (all *p* < 0.05). Antidepressant use was of borderline statistical significance (*p* = 0.067, AOR = 2.3 (95% CI: 0.94, 5.78), adjusting for all variables listed above and was not retained in the final model. Among all variables in the final model for hypertension (normotensive vs. hypertensive), the absolute value of spearman correlation coefficients were all less than or equal to 0.28, with spearman correlation coefficients greatest between female sex and quartile 4 of 25(OH)D_3_ (rho = −0.25, *p* = <0.001, and between no physical activity and unknown parental status of hypertension (rho = +0.19, *p* = 0.001), data not shown.

In the model of hypertension (normotensive vs. systolic and diastolic hypertension), stepwise selection retained female sex, body mass index, physical activity, anti-depressant use, smoking status, number of days from summer solstice, and two vitamin D biomarkers (1,25(OH)D_3_ and 24,25(OH)_2_D_3_). After inclusion of parental history of hypertension in the model, the variables for sex, body mass index, physical activity, anti-depressant use and 1,25(OH)_2_D_3_ and 24,25(OH)_2_D_3_ remained statistically significant and were therefore retained in the final model (all *p* < 0.05). Among all variables in the final model for hypertension, the absolute value of spearman correlation coefficients were all less than 0.30 in magnitude with the greatest correlation occurring between female sex and systolic and diastolic hypertension (rho = −0.30).

### Final model results

The odds of hypertension were significantly elevated among individuals reporting both parents having a history of hypertension (AOR = 4.5, 95% CI: 1.70–11.76), following adjustment for sex, body mass index, physical activity, and plasma concentration of 25(OH)D_3_ (Table 3). Overall, these variables explained approximately 21% of the variability in hypertensive (versus normotensive) status (*R*^2^ = 0.210). The odds of systolic hypertension (versus no systolic hypertension) was also significant among individuals reporting both parents having a history of hypertension (AOR = 7.1, 95% CI: 2.82, 17.66), following adjustment for sex, body mass index, physical activity, anti-depressant use and plasma concentrations of 1,25(OH)_2_D_3_ and 24,25(OH)_2_D_3_. Overall, these variables explained about 27% of the variability in systolic hypertension *R*^2^ = 0.267). In the diastolic hypertension model was significant among those overweight, antidepressant use, and concentrations of 25(OH)D_3_ and 24,25(OH)_2_D_3_. There was no evidence of significant lack of fit of either logistic regression model (H-L *p-*value = 0.841 and 0.269, respectively). Self-reported dual parental history of hypertension was also significantly associated with elevated blood pressure (v. normotension), after adjustment for sex, body mass index, physical activity, and plasma concentration of 25(OH)D_3_ (Supplemental Table 1).

**Table 3:** Adjusted odds of hypertension, systolic, and diastolic hypertension, young African American and European American Adults, Centre County Pennsylvania, 2006–2016.

	Hypertension (Stage I and II) v. Normotension			Systolic blood pressure (>120 mmHg)			Diastolic blood pressure (>80 mmHg)		
	OR	95% CI	*p-*value	OR	95% CI	*p*-value	OR	95% CI	*p*-value
Parental hypertensive status									
Both parents- Normotensive	*Reference*	–	–	*Reference*	–	–	*Reference*	–	–
Both parents- Hypertension	4.5	1.70, 11.76	0.002	7.1	2.82, 17.66	<0.001	NS	NS	NS
Mother only- Hypertension	1.5	0.72, 3.16	0.276	1.5	0.68, 3.20	0.304	NS	NS	NS
Father only-Hypertension	1.6	0.85, 3.06	0.140	1.7	0.86, 3.29	0.133	NS	NS	NS
Unknown parent status	1.9	0.37, 10.09	0.419	3.7	0.64, 20.43	0.144	NS	NS	NS
Sex									
Female	0.3	0.16, 0.49	<0.001	0.1	0.07, 0.26	<0.001	0.5	0.31, 0.85	0.009
Male	*Reference*			*Reference*			*Reference*	–	–
Body mass index (kg/m^2^)									
Underweight	2.1	0.36, 11.72	0.419	3.9	0.61, 24.86	0.147	1.8	0.32, 10.15	0.498
Normal	*Reference*	–	–	*Reference*	–	–	*Reference*	–	
Overweight	2.5	1.39, 4.43	0.002	1.8	0.96, 3.21	0.065	1.6	0.89, 2.78	0.116
Obese	7.3	3.31, 16.00	<0.001	7.3	3.38, 15.68	<0.001	2.6	1.35, 5.17	0.005
* P-*trend			<0.001			<0.001			<0.001
Physical activity frequency									
None	2.5	1.22, 5.27	0.013	2.2	1.04, 4.79	0.038	NS	NS	NS
A few times per month	2.1	1.05, 4.16	0.035	2.5	1.26, 5.05	0.009	NS	NS	NS
1–3 days per week	1.6	0.80, 3.20	0.182	2.0	0.97, 4.22	0.059	NS	NS	NS
4–6 days per week/every day	*Reference*	–	–	*Reference*	–	–	*Reference*	–	–
* P-*trend			0.010			0.014			NS
Antidepressant use (current)									
Yes	NS	NS	NS	3.5	1.38, 8.91	0.008	2.5	1.08, 5.79	0.033
No	*Reference*	–	–	*Reference*	–	–	*Reference*	–	–
Coffee intake (>1 cup per week)									
Yes	NS	NS	NS	NS	NS	NS	0.5	0.31, 0.96	0.036
No	*Reference*	–	–	*Reference*	–	–	*Reference*	–	–
25(OH)D_3_ (nmol/L)^a^									
7.7 to < 38.9	*Reference*	–	–	*Reference*	–	–	*Reference*	–	–
38.9 to < 63.4	2.5	1.26, 4.85	0.008	NS	NS	NS	2.2	1.07, 4.47	0.031
63.4 to < 116.4	1.3	0.62, 2.75	0.488	NS	NS	NS	1.8	0.67, 4.84	0.244
116.4 to 249.6	1.5	0.65, 3.58	0.338	NS	NS	NS	3.67	1.02, 13.22	0.046
* P-*trend			0.834			NS			0.028
1,25(OH)_2_D_3_ (pmol/L)									
35.5 to < 76.1	*Reference*	–	–	*Reference*	–	–	*Reference*	–	–
76.1 to < 96.0	NS	NS	NS	1.7	0.87, 3.52	0.105	NS	NS	NS
96.0 to < 116.4	NS	NS	NS	1.5	0.70, 3.27	0.284	NS	NS	NS
116.4 to 249.6	NS	NS	NS	2.4	1.07, 5.31	0.031	NS	NS	NS
* P-*trend			NS			0.048			NS
24,25(OH)_2_D_3_									
0.5 to < 3.5	*Reference*			*Reference*					
3.5 to < 7.6	NS	NS	NS	2.7	1.37, 5.15	0.004	1.5	0.73, 2.97	0.278
7.6 to < 12.9	NS	NS	NS	1.7	0.84, 3.62	0.136	0.7	0.27, 1.88	0.492
12.9 to 74.4	NS	NS	NS	0.7	0.25, 1.96	0.500	0.2	0.04, 0.66	0.012
* P-*trend			NS			0.364			0.260
H-L goodness of fit *P-*value			0.841			0.643			0.924
*R*^2^	0.210			0.267			0.112		

Each model adjusted for all variables shown in the table. *R*^2^ calculated using linear regression using SAS Proc Rec.

*NS* not statistically significant and excluded from the mode.

^a^ Quartile cut-offs determined according to the distribution among normotensive individuals in the study. Both models include parental hypertension status, sex, body mass index, physical activity.

## Discussion

Self-reported dual parental history of hypertension was positively associated both with elevated blood pressure, overall hypertension, and systolic hypertension among healthy young adult participants (ages 18–35). The magnitude of association with both parents having hypertension was substantial, ranging from 4.5-fold for overall hypertension to 7.1-fold for systolic hypertension. Parental hypertension status was not associated with elevated diastolic blood pressure. Our results are similar to previous studies, which report a 2.2-fold to 11-fold elevated risk [23, 24]. Forty percent of healthy young adult volunteers on this university campus were classified as either having elevated blood pressure or hypertension, based upon the 2017 ACC/AHA Hypertension Guidelines classification. Among those found to be hypertensive, nearly all (90.7%) were unaware of their status. Family history has long been known to be a risk factor for hypertension [4, 24–27] and only about 2% of participants in this study reported not knowing their family history. The lack of awareness of self-status of hypertension, in spite of knowing parental status, underscores a gap in clinical knowledge translation. This study was conducted among volunteers and therefore may not be representative of either the campus population or university students in general. Nationwide, young adults ages 18–39 years old in the US report a prevalence of self-awareness of hypertension from 52.1% to 74.7% in 1999–2000 and 2013–2015, respectively [2]. Nonetheless, the lack of awareness among volunteers alone is noteworthy and suggests that our efforts to inform students of their status as part of the study protocol participation were worthwhile.

To date, only a handful of studies have examined vitamin D and family history as risk factors for cardiovascular disease among young adults. Two other studies report an increased likelihood of hypertension among adolescents with at least one hypertensive parent, compared to adolescents that do not have a hypertensive parent [28, 29]. In a study conducted among adolescents aged 13–19 years old within the Korean National Health and Nutrition Examination Survey, those with a family history of hypertension had a three-fold risk of high blood pressure [30]. Bloetzer et al. conducted a study of 5207 children between the ages of 10–14 in Switzerland [28] wherein children with parents with hypertension had a two-fold risk of hypertension, compared to children that had parents without a history of hypertension. The prevalence of isolated systolic hypertension (ISH) among young adults ages 18–39 is low (less than 3%) in the US, although ISH prevalence in this age group has roughly tripled since 1988 [31]. Our study was not sufficiently large enough to investigate ISH, however, we did find evidence that dual parental family history is strongly associated (~7-fold) with systolic hypertension (>120 mmHg), regardless of diastolic blood pressure level. To our knowledge, no previous studies have adjusted for vitamin D status in the investigation of risk associated with a family history of hypertension.

Circulating blood concentrations of 25(OH)D_3_ have a moderately high heritability (heritability index range 0.69–0.86) according to adolescent twin studies [32]. We therefore hypothesized that, because of the heritability of circulating vitamin D concentrations, that the association with family history would be muted upon adjustment for vitamin D status. However, this was not the case. Our results are consistent with previous studies reporting increased risk of hypertension according to male sex, low physical activity, higher body mass index, antidepressant use and blood concentrations of 25(OH)D_3_ [33–36]. The association we observed with 25(OH)D_3_ was not linear in relation to hypertension. Notably, although West African genetic ancestry is a well-known determinant of 25(OH)D_3_ concentrations [16] it was not associated with hypertension in this study. Non-linear associations with 25(OH)D_3_ and hypertension have been previously reported in meta-analyses [37]. In addition, oral contraceptive use can increase 25(OH)D_3_ concentrations by as much as 25% in both observational studies and dosing trials (11). The association with the dihydroxy-vitamin D metabolites (1,25(OH)_2_D_3_ and 24,25(OH)_2_D_3_) with systolic hypertension (and not overall hypertension) is interesting to note and might be explained by the fact that the kidney is the body’s major source of hydroxylation for these two circulating metabolites, although only the linear test for trend for 1,25(OH)_2_D_3_ was not statistically significant. Chronic kidney disease is more strongly associated with systolic blood pressure than diastolic blood pressure [38]. It is also known that reduced production of these dihydroxy-vitamin D metabolites is a hallmark of CKD progression [39].

### Strengths and limitations

This study is one of the first to examine vitamin D and family history together as risk factors for hypertension among young adults following the release of the new ACC/AHA Hypertension Guidelines. The strengths of this study include, use of a clinical research center with personnel trained in nutritional epidemiology and the NHANES measurement protocols, inclusion of a validated panel of West African Genetic Ancestry proportion as an unbiased way to control for possible influences of race/ethnicity, and use of a validated LC/MS-MS assay for determination of multiple vitamin D biomarkers within a study designed to assess vitamin D status [16]. Limitations include the possible misreported family history of hypertension, although if the misreporting is randomly distributed, this reporting error would be expected to depress the OR. Finally, many of the participants in the present study were young adult volunteers attending college, and therefore the findings of this study may not be generalizable to other groups of young adults in the United States. In addition, we did not find a strong association with cigarette smoking, as observed in previous studies. This may be due to the younger age of our study population and the low overall prevalence of smoking (~13%).

The findings of this study suggest a strong influence of the dual parental history of hypertension in the development of elevated blood pressure, hypertension, and systolic hypertension among otherwise healthy young adults. Parental history appears to be independent of sex, physical activity, body mass index, and vitamin D status. The overall prevalence of hypertension and elevated blood pressure (~40%) and the combined lack of self-awareness of hypertensive status among well-educated healthy young adults underscores the need for targeted primary and secondary prevention efforts.

### Summary table

#### What is known about the topic?

- Young adults are often unaware of their hypertension status and less likely to have controlled hypertension.
- Family history of hypertension is a risk factor for hypertension among children and young adults.
- Low plasma vitamin D levels are a risk factor for developing cardiovascular diseases such as stroke and myocardial infarction.

#### What this study adds?

- Dual parental history of hypertension is a risk factor for the development of elevated blood pressure, hypertension, and systolic hypertension among healthy young adults.
- The lack of awareness of hypertension status, in spite of knowing parental status, underscores a gap in clinical knowledge translation.
- The study findings highlighted an independent association with dual parental history and hypertension status, regardless of vitamin D status.

## Disclaimer

The findings of this study have not been published previously and is not under review at another journal.

## Supplementary information

Supplemental File
