# Supplementary material for: Vitamin D and family history of hypertension in relation to hypertension status among college students
Source: J Hum Hypertens. 2021 Jul 20;36(9):839–45. doi: 10.1038/s41371-021-00577-6 (PMC9467912; doi:10.1038/s41371-021-00577-6)
Supplement: Supplementary file 1 — Supplemental File [file 41371_2021_577_MOESM1_ESM.docx]

Supplemental Table 1: Adjusted Odds of Hypertension and Elevated Blood Pressure, Young African American and European American Adults, Centre County Pennsylvania, 2006-2016

|  | Hypertension v. Normotension | | | Elevated Blood Pressure v. Normotension | | |
| --- | --- | --- | --- | --- | --- | --- |
|  | AOR | 95% CI | p-value | AOR | 95% CI | p-value |
| Parental Hypertensive Status |  |  |  |  |  |  |
| Both Parents Normotensive | *Reference* | --- | --- | *Reference* | --- | --- |
| Both Parents Hypertensive | 4.5 | 1.70, 11.76 | 0.002 | 12.3 | 3.29, 46.05 | <0.001 |
| Mother Hypertensive Only | 1.5 | 0.72, 3.16 | 0.276 | 2.5 | 0.78, 8.24 | 0.121 |
| Father Hypertensive Only | 1.6 | 0.85, 3.06 | 0.140 | 1.5 | 0.50, 4.72 | 0.458 |
| Unknown Parent Status | 1.9 | 0.37, 10.09 | 0.419 | 3.8 | 0.33, 43.97 | 0.287 |
| Sex |  |  |  |  |  |  |
| Female | 0.3 | 0.16, 0.49 | <0.001 | 0.2 | 0.06, 0.40 | <0.001 |
| Male | *Reference* | --- | --- | *Reference* | --- | --- |
| Body Mass Index (kg/m^2^) |  |  |  |  |  |  |
| Underweight | 2.1 | 0.37, 11.72 | 0.419 | NC | NC | 0.983 |
| Normal | *Reference* | --- | --- | *Reference* | --- | --- |
| Overweight | 2.5 | 1.39, 4.43 | 0.002 | 1.1 | 0.42, 2.98 | 0.829 |
| Obese | 7.3 | 3.31 16.00 | <0.001 | 5.6 | 1.70, 18.24 | 0.005 |
| *P-*trend |  |  | <0.001 |  |  | <0.001 |
| Physical Activity Frequency |  |  |  |  |  |  |
| None | 2.5 | 1.22, 5.27 | 0.013 | 1.9 | 0.62, 6.03 | 0.259 |
| A few times per month | 2.1 | 1.05, 4.16 | 0.035 | 3.2 | 1.23, 8.47 | 0.017 |
| 1-3 days per week | 1.6 | 0.80, 3.20 | 0.182 | 0.2 | 0.02, 1.40 | 0.100 |
| 4-6 days per week/Every day | *Reference* | --- | --- | *Reference* | --- | --- |
| *P-*trend |  |  | 0.010 |  |  | 0.058 |
| 25(OH)D_3_^*^ (nmol/L) |  |  |  |  |  |  |
| 7.7 to < 38.9 | *Reference* | --- | --- | *Reference* | --- | --- |
| 38.9 to < 63.4 | 2.5 | 1.26, 4.85 | 0.008 | 1.6 | 0.57, 4.22 | 0.390 |
| 63.4 to < 116.4 | 1.3 | 0.62, 2.75 | 0.488 | 1.6 | 0.48, 5.04 | 0.457 |
| 116.4 to 249.6 | 1.5 | 0.65, 3.58 | 0.338 | 1.4 | 0.35, 5.58 | 0.630 |
| *P-*trend |  |  | 0.834 |  |  | 0.605 |
|  |  |  |  |  |  |  |
| H-L Goodness of Fit *P-*value |  |  | 0.841 |  |  | 0.269 |
| R^2^ | 0.223 |  |  | 0.210 |  |  |
|  |  |  |  |  |  |  |

Each model adjusted for all variables shown in the table.

*quartile cut-offs determined according to the distribution among normotensive individuals in the study. Both models include parental hypertension status, sex, body mass index, physical activity.

NC = Not calculated due to small sample size in this category.

R^2^ calculated using linear regression using SAS Proc Reg.

**Wave 3**

**(2013-2016)**

n=48

**Wave 2**

**(2009)**

n=303

**Wave 1**

**(2006-2007)**

n=103

**Master Dataset**

n=457

**Exclusions**: hypotensive (n=13); race/ethnicity other than black/white (n=7); diabetes (n=4); antihypertensives (n=2); no biomarker information (n=36); incomplete BMI (n=1); missing physical activity history (n=1) missing smoking history (n=1)

**Analytic Subset**

n=396

**Normal Blood Pressure**

n=238

**Hypertensive**

n=120

**Elevated Blood Pressure**

n=38

**Supplemental Figure 1.** Flow Chart of Data Collection Periods and Exclusion Criteria
